# Clinical characteristics and outcome of very old (≥90 years) critically ill patients with need for intensive care after surgical intervention

**DOI:** 10.3389/fmed.2025.1509337

**Published:** 2025-05-09

**Authors:** Jöran Lücke, Franziska Stallbaum, Rikus Daniels, Pauline Theile, Jakob Izbicki, Anastasios D. Giannou, Stefan Wolter, Anna Duprée, Oliver Mann, Jakob Müller, Stefan Kluge, Matthias Reeh, Kevin Roedl

**Affiliations:** ^1^Department of General, Visceral and Thoracic Surgery, University Medical Center Hamburg-Eppendorf, Hamburg, Germany; ^2^Section of Molecular Immunology and Gastroenterology, Department of Medicine, University Medical Center Hamburg-Eppendorf, Hamburg, Germany; ^3^Hamburg Center for Translational Immunology, University Medical Center Hamburg-Eppendorf, Hamburg, Germany; ^4^Department of Medicine, University Medical Center Hamburg-Eppendorf, Hamburg, Germany; ^5^Department of Intensive Care, University Medical Center Hamburg-Eppendorf, Hamburg, Germany; ^6^Department of Anesthesiology, Tabea Hospital, Hamburg, Germany

**Keywords:** critically ill, elderly, older, very old, outcome, visceral surgery, thoracic surgery

## Abstract

**Background:**

Due to increasing life expectancy and decreasing morbidity, society is aging. Especially the group of very old patients (≥90 years) is currently increasing significantly. This leads to increasing numbers of very old patients requiring visceral and thoracic surgery. However, due to the multimorbidity of this cohort, postoperative intensive care is often required. Currently, insufficient data are available regarding the treatment and therapy of very old surgical critically ill patients. Therefore, this study aimed to investigate factors associated with early postoperative mortality to identify patients at risk.

**Methods:**

A retrospective cohort study was performed using clinical and sociodemographic data of all very old patients admitted to the Department of Intensive Care Medicine after visceral or thoracic surgery at the University Medical Center Hamburg-Eppendorf, between January 2008 and April 2019. Univariate comparisons and multivariate regression analysis were performed to identify factors associated with early postoperative death.

**Results:**

In total, 84 patients were included in the analysis. In this cohort, 27 (32.14%) died within 28 days after admission to the intensive care unit (ICU). Comparisons between these two groups show no association between the specifics of the operative procedure with early postoperative death. However, the living situation before hospital admission was shown to be significantly different between those patients who survived and those who died early after postoperative care. We further show an association of early postoperative death with preexisting cardiac diseases, increased creatinine levels, and high Simplified Acute Physiology Score II (SAPS II) upon admission to ICU.

**Conclusion:**

The SAPS II score and creatinine level upon admission could be identified as predictors of survival and may be beneficial for risk stratification in the critically ill patient cohort of the very old.

## Introduction

Society is aging due to the emergence of extensive medical achievements and reduced mortality of nearly all age groups (1). Consecutively, an increasing number of older patients undergo surgery and require extended postoperative surveillance in intensive care units (ICU) (2, 3). For example, in one study, over 13% of ICU admissions between 2001 and 2005 could be assigned to very old patients, meaning patients being 80 years of age or older, with the projected trend continuously increasing (4). Despite the achievements of intensive care, admission to an ICU is associated with great stress for very old patients and their relatives. Considering the limited resources of the ICU and the high post-operative mortality (5), surgery and postoperative admission in an ICU have to be considered carefully in this cohort. In line with this, many physicians remain skeptical of the beneficial effects of ICU admissions of very old patients (6). Moreover, a recent interventional study found that a systematic admission of very old patients to the ICU did not significantly reduce their mortality (7). Currently, no guidelines exist for the very old critically ill patients with a need for intensive care after surgical intervention. Thus, careful evaluation of which patients might benefit from postoperative ICU admission is required, and identifying determinants of early postoperative death is essential.

The process of aging is associated with a decline in many bodily and cognitive functions (8). Therefore, it is no surprise that an increase in mortality of very old patients in the ICU compared to a younger patient cohort could be detected (9). At least two studies have so far reported that a major predictor for postoperative survival of very old patients in the ICU is the occurrence of a medical complication (10, 11). Another factor that seems to determine the outcome of very old patients is whether an elective or emergency surgery was performed (12). This recent study further showed that very old patients had significantly higher survival and lower Sequential Organ Failure Assessment (SOFA) score if they underwent elective surgery (12). However, studies that evaluate prognostic factors of very old patients being admitted to the ICU after surgery are currently scarce. Thus, in this study, the primary aim was to investigate associations of established laboratory findings and scores with early postoperative death among the over 90-year-old patients that received visceral or thoracic surgery and were subsequently admitted to the ICU.

## Materials and methods

### Sample

This present study presents data obtained from a retrospective study that was conducted at the ICUs of the University Medical Center Hamburg-Eppendorf. It includes 1108 over 90-year-old patients that were admitted to the ICU between January 2008 and April 2019. The analytic sample is comprised of 84 patients admitted because of a visceral- or thoracic surgical main diagnosis that required surgery. These patients were identified in a retrospective fashion using the electronic medical archive of the hospital. All methods were carried out in accordance with relevant guidelines and regulations. The Ethics Committee of the Hamburg Chamber of Physicians approved the study (Number 2022-300219-WF). Due to the study’s retrospective nature and anonymized data collection, the need for informed consent was waived by the Ethics Committee of the Hamburg Chamber of Physicians in accordance with national guidelines. This analyzed dataset is not publicly available but may be provided by the corresponding author Jöran Lücke upon reasonable request.

### Variables

Basic sociodemographic variables including age (in years), sex, body mass index (in kg/m^2^), the living situation before admission, preexisting medical conditions, and postoperative parameters in ICU, such as the need for catecholamines, ventilation or laboratory values were included in the analysis. Furthermore, surgical specifications such as the preoperative diagnosis, intraoperative procedures, and durations were collected from the electronic medical charts and surgical protocols. The SAPS II and SOFA scores as well as the Charlson Comorbidity Index (CCI) were calculated according to published formulas (13–15). Preexisting medical conditions were defined as follows: Cardiac diseases included status after a heart attack, heart insufficiency, atrial fibrillation, and aortic stenosis; vascular included peripheral artery occlusive diseases and cerebrovascular occlusive diseases; oncologic diseases included solid malignant tumors including metastasis and all malignant liquid tumors.

### Outcome measurement

Survival post-admission to the ICU (days) was dichotomized, defining early postoperative death as survival of ≤ 28 days after admission to the ICU.

### Statistics

First, the present study cohort was divided into two groups according to their 28-day survival. Second, univariate comparisons were made using a two-sample Wilcoxon rank-sum for metric, and chi2 or fisher’s exact test for logistic variables. Subsequently, logistic regression analyses were performed using survival data as the dependent variable, and statistically significantly different clinical and sociodemographic factors as independent variables. Model estimators for logistic models are provided as likelihood quotient tests, for logistic models as a coefficient of determination. Statistical significance was defined using α = 0.05. Statistical analysis was carried out using Stata 17.0 (Stata Corp, College Station, TX, USA).

## Results

### Characteristics of the study population

Out of 1108 ≥ 90-year-old patients admitted to the ICUs of the University Medical Center Hamburg-Eppendorf between January 2008 and April 2019, 84 patients with a visceral- or thoracic surgical main diagnosis that required surgical treatment were identified. Of these 84 patients, 27 patients (32.14%) died within 28 days of admission to the ICU. The baseline characteristics of these patients and the other 57 patients that survived longer than 28 days are displayed in Table 1, an overview on missing data is provided in Supplementary Table 1. Of note, out of the 27 patients that died within the first 28 days after admission, 9 patients died directly on ICU, while 16 patients died on the regular ward afterward. Only 2 patients in this cohort were discharged and died in other locations. Out of the surviving 57 patients, 9 patients died during the next consecutive year (data not shown). Interestingly, these two patient groups significantly differed in their pre-admission living situation. Additionally, patients surviving longer than 28 days had a lower percentage of cardiac pre-existing conditions but did not differ in Charlson Comorbidity Index (CCI) at admission.

**TABLE 1:** Baseline data and demographic characteristics of the cohort stratified by survival status at 28 days.

Variables	Total *N* (%) / median (IQR) (*N* = 84)	Survival > 28 days *N* (%) / median (IQR) (*N* = 57)	Exitus within 28 days *N* (%) / median (IQR) (*N* = 27)	*P*-value univariate comparison
**Sociodemographics and health at admission**				
Age (years)	92.3 (91–94)	92.2 (91–94)	92.3 (91–94)	0.979
Gender (ref: male)	60 (71.4%)	40 (70.2%)	20 (74.1%)	0.712
Body mass index (kg/m^2^)	22.9 (21.3–25.0)	22.9 (21.5–25.0)	22.0 (21.2–24.2)	0.554
Living situation before admission				0.004
At home	55 (65.5%)	44 (77.2%)	11 (40.7%)	
Nursing home	8 (9.4%)	4 (7.0%)	4 (14.8%)	
Assisted living facility	21 (24.7%)	9 (15.8%)	12 (44.4%)	
Limitation of therapy (ref: none)	16 (19%)	4 (25%)	12 (75%)	<0.001
Charlson Comorbidity Index	2 (1–3)	2 (1–3)	2 (1–3)	0.176
**Preexisting medical condition**				
Cardiac disease (ref: no)	42 (50%)	24 (42.1%)	18 (66.7%)	0.035
Hypertension (ref: no)	56 (66.7%)	38 (66.7%)	18 (66.7%)	1.000
Arterial obstructive disease (ref: no)	15 (17.9%)	9 (15.8%)	6 (22.2%)	0.472
Diabetes mellitus (ref: no)	8 (9.5%)	6 (10.5%)	2 (7.4%)	1.000
Oncological disease (ref: no)	31 (36.9%)	20 (35.1%)	11 (40.7%)	0.616
Nephrological disease (ref: no)	16 (19.1%)	8 (14.0%)	8 (29.6%)	0.089
Dementia (ref: no)	15 (17.9%)	9 (15.8%)	6 (22.2%)	0.472
**Surgical intervention**				
Planned ICU admission (ref: no)	36 (42.4%)	28 (49.1%)	8 (29.6%)	0.092
Duration of surgery (min)	106 (68–175)	105 (64–165)	110 (75–176)	0.7663
Secondary surgery (ref: no)	17 (20.2%)	14 (24.6%)	3 (11.1%)	0.123
Postoperative bleeding (ref: no)	2 (2.4%)	2 (3.5%)	0 (0.0%)	1.000
**Basic postoperative parameters in ICU**				
Need for postoperative catecholamines (ref: no)	40 (47.1%)	22 (38.6%)	18 (66.7%)	0.016
Need for postoperative ventilation (ref: no)	26 (30.6%)	13 (22.8%)	13 (48.1%)	0.019
Enteral nutrition (ref: no)	76 (89.4%)	55 (96.5%)	21 (77.8%)	0.006
Length of stay in ICU (days)	2 (1–3)	2 (1–3)	2 (1–6)	0.446
Length of stay in hospital (days)	13 (8–19)	14 (9–19)	11 (6–19)	0.083

Furthermore, patients that survived during the first 28 days required catecholamines and mechanical ventilation less often upon ICU admission and had a higher rate of postoperative enteral nutrition. Unsurprisingly, the percentage of patients that decided to limit therapy before or during their stay in the ICU was also significantly higher in the group of non-survivors.

When comparing the parameters of mechanical ventilation in the 26 patients that arrived with it, we found that patients that survived the consecutive 28 days had a lower positive end-expiratory pressure (PEEP) and FiO_2_ at ICU admission (Supplementary Table 2).

### Preoperative diagnosis and intraoperative procedures

Next, we investigated the diagnosis, and thus, the surgical indications that led to surgery in ≥90-year-old patients. The most common diagnosis of these patients was ileus (35.7%), carcinoma (30.9%), bleeding (16.7%), perforations (13.1%), and cholecystitis (13.1%) (Supplementary Table 3).

We then compared the intraoperative procedures that were performed during surgery. Most abundantly, colon resection was performed, followed by cholecystectomy (Supplementary Table 4). There were no differences in operative procedures between patients that survived the consecutive 28 days and the patients that died within that time span.

### Laboratory values and scores upon admission and at consecutive timepoints

We next evaluated whether clinical scores or laboratory values that were determined upon admission differed in patients with or without survival during 28 days upon ICU admission. Indeed, patients that survived this timespan had significantly lower SAPS II and SOFA scores, higher urine production, pH, bicarbonate, and base excesses, as well as lower lactate levels in their arterial blood gas (Table 2). Moreover, the venous creatinine and GOT levels, as well as the INR were significantly lower in the patient cohort with a survival greater than 28 days. Of note, only three patients received hemodialysis during their stay on the ICU, with one of these patients surviving more than 28 days and two patients that did not (data not shown). All these three patients needed hemodialysis as outpatients and thus, before their initial admission on ICU.

**TABLE 2:** Laboratory values and scores upon admission stratified by survival status at 28 days.

Upon admission	Physiological range according to (16) and (17) PA	Total *N* (%) / median (IQR) (*N* = 84)	Survival > 28 days *N* (%) / median (IQR) (*N* = 57)	Exitus within 28 days *N* (%) / median (IQR) (*N* = 27)	*P*-value univariate comparison
**Daily scores and clinical characteristics**					
SAPS II (score)		36 (29–50)	32 (28–41)	49 (38–60)	0.001
SOFA (score)		3 (1–7)	1 (0–4)	5 (3–9)	0.0003
Urine production (ml/24 h)		425 (180–790)	532 (340–870)	260 (0–500)	0.003
**Arterial laboratory values**					
pO_2_ (mmHg)	80–100	93.1 (77–124)	92.2 (76.1–119)	106 (82–143)	0.126
pCO_2_ (mmHg)	35–45	40.4 (31.7–43.9)	41.4 (36.6–44.6)	38.5 (31.7–43.9)	0.148
pH	7.38–7.44	7.36 (7.33–7.41)	7.38 (7.33–7.41)	7.33 (7.27–7.38)	0.020
Bicarbonate (mmol/l)	21–28	22.4 (20.4–24.4)	23.3 (21.6–24.9)	20.6 (17.6–22.3)	0.0001
Base excess (mmol/l)	−4 to +2	−2.7 (−5.3 to 0)	−1.4 (−3.55 to −0.55)	−4.7 (−8.1 to −2.7)	0.0002
Lactate (mmol/l)	0.6–1.7	1 (0.8–1.6)	0.9 (0.65–1.2)	1.8 (1.1–2.4)	0.0001
**Venous laboratory values**					
Hemoglobin (g/dl)	12.0–17.5	11.0 (9.8–12.3)	10.9 (9.9–12.5)	11.1 (9.5–11.9)	0.885
Leukocyte count (×10^9^/l)	4.5–11.0	11.2 (7.8–14.2)	11.9 (8.4–16.3)	11.9 (8.4–16.3)	0.321
Thrombocyte count (×10^9^/l)	150–350	239 (189–281)	240 (190–281.5)	238 (179–278)	0.573
Creatinine (mg/dl)	<1.5	0.9 (0.7–1.5)	0.8 (0.66–1.1)	1.5 (0.8–1.7)	0.002
Bilirubin (mg/dl)	0.3–1.0	0.7 (0.5–1)	0.7 (0.4–0.9)	0.7 (0.6–1.1)	0.248
Aspartate transaminase (AST) (U/l)	0–35	29 (20–72)	23 (17–46)	53 (24–129)	0.027
Alanine transaminase (ALT) (U/l)	0–35	17 (11–31)	16 (10–30)	18 (13–34)	0.323
Gamma-glutamyl transferase (GGT) (U/l)	1–94	52 (29–126)	45 (25–60)	60 (41–132)	0.161
Lactate dehydrogenase (LDH) (U/l)	100–190	241 (206–319)	219 (196–251)	274 (241–406)	0.110
C-reactive protein (CRP) (mg/l)	0.08–3.10	53 (12–96)	46 (9–90)	57 (29–98)	0.228
International normalized ratio (INR)	0.8–1.1	1.1 (1.0–1.2)	1.1 (1.0–1.2)	1.2 (1.1–1.3)	0.003

We also investigated laboratory values at two consecutive time points, specifically, 24 and 48 h upon admission to ICU. Of note, the patient sample size became smaller with increasing observation timespan, since patients were either discharged from the ICU or died. In line with the previous observations, we found that after 24 h, higher pH, bicarbonate, and base excess levels, as well as lower lactate levels, creatinine levels, GOT levels, and a lower INR, in addition to a lower rate of ventilated patients and patients with need of catecholamines, were still present (Supplementary Table 5). Additionally, lower GGT levels and a reduced SOFA score could be detected in patients that survived the first 28 days. Finally, 48 h after admission to ICU, survivors still had significantly higher bicarbonate and base excess levels, and significantly lower lactate and creatinine levels (Supplementary Table 6).

### Multivariate regression analysis

To determine factors associated with early postoperative death, we finally performed multivariate regression using complete cases including sociodemographic, as well as laboratory values that were shown to be significantly different between both groups in the univariate analysis. We found that the decision to limit therapy, preexisting medical conditions of the heart, SAPS II score upon admission, and creatinine levels were associated with early postoperative death (Table 3).

**TABLE 3:** Logistic regression analysis of selected variables stratified by survival status after 28 days.

Non-survival after 28 days	Odds-ratio	95% confidence interval		*P*-value
**Sociodemographics, health, and medical conditions**				
Living situation before admission (ref: at home)				
Assisted living facility	1.82	0.41	80.37	0.31
Nursing home	1.36	0.11	16.40	0.25
Limitation of therapy	166.17	3.87	7136.86	0.008
Preexisting cardiac disease	29.85	1.16	768.01	0.040
**Clinical characteristics at admission**				
Need for postoperative catecholamines	1.23	0.03	36.56	0.904
Need for postoperative ventilation	0.21	0.00	35.45	0.550
Enteral nutrition	0.14	0.00	4.40	0.268
SAPS II (score)	1.14	1.02	1.28	0.030
SOFA (score)	0.66	0.27	1.62	0.366
Urine (ml/24 h)	0.99	0.99	1.00	0.167
**Arterial laboratory values at admission**				
pH	382803.5	0.00	2.07 × 10^11^	0.181
Lactate (mmol/l)	2.07	0.59	7.34	0.258
Base excess (mmol/l)	0.99	0.76	1.27	0.919
**Venous laboratory values at admission**				
Creatinine (mg/dl)	14.52	1.68	125.69	0.015
International normalized ratio (INR)	170.13	0.39	74127.7	0.098
Model estimator	Number of observations = 73 Likelihood ratio = 54.42 Prob > chi2 = 0.0000			

Finally, we performed a multivariate analysis for the 1-year survival. Among the variables described above, the decision to limit therapy, preexisting medical conditions of the heart, and creatinine levels were also associated with survival after 1 year (Supplementary Table 7).

## Discussion

The present study aimed to investigate predictive factors that were associated with the postoperative survival of very old patients with visceral or thoracic surgery admitted to the ICU. We found that several factors were significantly different between survivors and non-survivors in the first 28 days. Among these factors were not only the living situation before hospital admission and cardiac pre-existing medical conditions but also ICU-specific procedures. Furthermore, our multivariant analysis revealed that the decision to limit therapy, increased creatinine level, and high SAPS II score at admission were found to be independent predictive factors for elevated mortality, that might be used as predictive factors on ICU wards in the future.

### Pre-admission living situation and outcome of intensive care treatment

Our study found that the pre-admission living situation was significantly different in the group that survived the first 28 days, compared to the group that did not. Specifically, patients with a more independent living situation before admission were found more frequently in the group that survived 28 days. This observation could be corroborated by several other studies that investigated the mortality of patients without distinguishing between the reasons for admission. For example, a study from Denmark investigated all admitted patients to ICU and found that the pre-admission quality of life determined by different scores indeed predicted 30- and 90-day mortality in a way that patients with a better pre-admission quality of life survived longer (18). The same observation could be made from another study from Denmark (19), that included all patients that stayed longer than 48 h in the ICU ward. Since age greatly correlates with the pre-admission quality of life (20), it is especially important to compare the pre-admission quality of life between the same age groups. Indeed, higher functionality and quality of life in very old patients before admission to the ICU was also a significant factor for reduced mortality (21, 22). Of note, the inclusion age that defines patients as “very old” varies from study to study, with some studies including patients from the age of over 70 and onwards. Taken together, our observation concerning a cohort of surgical admissions of very old patients to the ICU falls in line with previous reports describing similar results, but without stratifying for admission diagnosis.

### Cardiac pre-existing medical conditions and outcome of intensive care treatment

Comorbidity is more than common in the cohort of very old patients (23) and is associated with severe mortality risk (24). This is especially evident during a stay in an ICU, during which comorbidity presents as one of the most determining factors for mortality (4, 25). This is also true for cardiovascular comorbidity, which majorly determines survival upon ICU admission in multiple studies, for example in the context of post-burn-treatment (26) or coronavirus disease 2019 (COVID-19) (27). Interestingly, in this study, we found that only cardiac pre-medical conditions, but not others (such as oncological, nephrological, or endocrinological) influenced mortality in our cohort of very old, surgical patients. This might be explainable by the distinctly increased cardiac peri- and postoperative risk in very old patients with cardiovascular comorbidity (28). Consecutively, intra- or postoperative cardiac events, such as myocardial infarction, present the second leading comorbidity in the very old in a recent study from Taiwan (29). Since the median length of stay in the ICU in our study was only 2 days, perioperative risk factors such as cardiovascular premedical conditions might be especially relevant to assess the mortality of our cohort compared to a non-surgical patient group.

### Mechanical ventilation, catecholamine therapy, and outcome of intensive care treatment

Our study furthermore revealed that the need for extended mechanical ventilation in the ICU was significantly elevated in the group of non-survivors. Similar observations were also made by other studies, underlining the benefit of non-invasive ventilation in the cohort of very old patients whenever possible (22, 30). However, while an invasive method of ventilation in non-surgical patients inevitably means an escalation in therapy with increased rates of complication, visceral and thoracic surgery patients need to be mechanically ventilated, at least for the duration of the surgical intervention. Nonetheless, a study found that prolonged mechanical ventilation after surgery in very old patients was correlated to increased mortality (31). Our study corroborates this finding, since patients that did not require mechanical ventilation in the ICU as they could be extubated before arrival presented an enhanced survival rate. Additionally, we could further stratify the mortality of ICU-ventilated patients according to their PEEP and FiO_2_ requirements upon arrival. Regarding catecholamine therapy, the results of studies are more conflicted. While some studies report that the requirement of catecholamine therapy is a negative prognostic factor in the treatment of very old patients in the ICU (32, 33), other studies did not make this observation (34). Thus, further studies with bigger cohorts are warranted to determine in which cases vasopressor therapy proves unbeneficial for the outcome of the very old patient.

### SAPS II score and outcome of intensive care treatment

Moreover, we found that the SAPS II score at admission was significantly higher in non-survivors according to our multivariate regression analysis. The SAPS II score is a commonly used score to calculate morbidity upon ICU admission using basic vital and laboratory findings from the patient (13). Thus, it is no wonder that many published studies equally found that the SAPS II score was associated with mortality in the cohort of very old patients being admitted to the ICU. For example, one study compared the predictive capacity of different scores in a cohort of patients being admitted to the ICU that were 80 years or older. Indeed, the SAPS II score predicted mortality best in comparison to other commonly used scores (35). Another study from France further corroborated the predictive power of the SAPS II score in a cohort of 85-year-old patients or older (36). Of note, other measurements specifically designed for elderly patients such as the Clinical Frailty Scale could have been used to determine not only the laboratory findings and vital signs, but also the functional status of the patients (37). Other studies already demonstrated that the Clinical Frailty Scale corresponded with survival on the ICU of elderly patients (38). However, this and other scores were not initially assessed on the ICU in our center so that it is not retrospectively possible to include this and other scales into our analysis. Together with our data, the preexisting reports might help reinforce this score as an early predictive variable for the risk of mortality in the group of very old patients in the ICU.

### Creatinine levels and outcome of intensive care treatment

Finally, our study revealed a statistically significant association between higher creatinine levels and higher mortality. In line with this, the group that did not survive 28 days after admission, showed overall reduced urine production. Elevated creatinine levels can have different causes, the two most common being acute kidney injury (AKI) and chronic kidney disease (CKD). Most importantly, AKI in the cohort of the very old in the ICU is associated with significantly elevated mortality (39). A recent study investigated risk factors for AKI in very old patients after abdominal surgery and found that, next to other reports, intraoperative hypotension or the usage of furosemide is associated with an increased risk for AKI (40). Thus, special attention should be given to early intra- and postoperative signs of AKI to be able to initiate appropriate countermeasures early. Likewise, patients with CKD and especially patients suffering from end-stage renal disease (ESRD) possess elevated mortality in the ICU (41). Moreover, CKD is a major risk factor for developing an AKI with the consequences discussed above (41). Therefore, elevated creatinine levels were established as a risk factor for very old patients in the ICU by previous studies (22), as could be corroborated by our study for the cohort of very old surgical patients in the ICU.

### Ethical aspects

ICU treatment remains expensive, and its admission capacity is limited, which comes especially apparent in times of emerging health crises and pandemics. Thus, it needs to be discussed whether access to ICU treatment should be limited in times of scarcity for groups that overwhelmingly do not to profit from such an admission. One parameter that is often discussed as a predictor for enhanced mortality on ICU is age. Indeed, there is great doubt among many physicians that elderly patients profit from ICU admissions (6), which was corroborated by a recent study (7). However, our study could confirm that a defined sub cohort of very old postoperative patients survived ICU, which clarifies that age alone might be an insufficient parameter to deny patients ICU care. Instead, while age might be among prognostic factors for short- and long-term survival after ICU admission, other factors should be taken into account as well, some of them being discussed above. However, these observations do not answer one of the most profound ethical questions, being to which end a statistical good prognosis of a patient if admitted to ICU should determine his/her chance of ICU admission in situations of scarcity. In these circumstances, other ethical considerations to maximize the distribution of beneficial resources are of high importance (42).

### Limitations of the study

Despite all strengths outlined above, our study has some limitations that need to be addressed. First, this is an investigation conducted at one single center. Therefore, patients were drawn from the surrounding urban area that the hospital is located in, and results might not be easily transferrable to patient cohorts in other settings. Second, this study is retrospective. This means that some parameters could not be assessed from all included patients, or could not be assessed at all, which might have proved beneficial for the readout. Third, the investigated cohort is rather small, requiring follow-up studies with bigger samples-sizes to corroborate the outlined findings. This is especially important for conclusions drawn from small subgroups, for example when regarding clinical parameters and laboratory values 48 h after ICU admission. Forth, this study cannot be transferred to the whole cohort of very old critically ill patients, as we only investigated patients that were admitted to the ICU. Thus, patients that were directly admitted to regular ward after surgery and patients, that did not receive any surgery due to their pre-operative critical condition or the decision to limit therapy are not reflected in this study, which renders a significant selection bias regarding the whole cohort of very old critically ill patients. Lastly, patients were included over a time span of roughly 10 years, opening up a small, but non-negligible possibility for chronological biases.

## Conclusion

Taken together, analyzing our displayed cohort of very old critically ill patients demonstrated that the SAPS II and creatinine levels upon admission could be identified as predictors of survival, even after multivariate analysis. Thus, they could aid in future risk stratification, helping to identify patients profiting from postsurgical intensive care admission.

## Data Availability

The raw data supporting the conclusions of this article will be made available by the authors, without undue reservation.
