# Supplementary material for: Clinical characteristics and outcome of very old (≥90 years) critically ill patients with need for intensive care after surgical intervention
Source: Front Med (Lausanne). 2025 May 9;12:1509337. doi: 10.3389/fmed.2025.1509337 (PMC12098617; doi:10.3389/fmed.2025.1509337)
Supplement: Supplementary file 1 [file Data_Sheet_1.docx]

**Supplementary tables**

|  | Number of missing values (% of the total cohort) |
| --- | --- |
| Age (years) | 0 (0) |
| Gender (ref: male) | 0 (0) |
| Body mass index (kg/m^2^) | 20 (23.8) |
| Living situation before admission | 0 (0) |
| Limitation of therapy  (ref: none) | 0 (0) |
| Charlson Comorbidity Index | 0 (0) |
| **Preexisting medical condition** | |
| Cardiac disease | 0 (0) |
| Hypertension | 0 (0) |
| Arterial obstructive disease | 0 (0) |
| Diabetes mellitus | 0 (0) |
| Oncological disease | 0 (0) |
| Nephrological disease | 0 (0) |
| Dementia | 0 (0) |
| **Surgical intervention** | |
| Elective surgery | 0 (0) |
| Duration of surgery | 2 (2.4) |
| Secondary surgery | 0 (0) |
| Postoperative bleeding | 0 (0) |
| **Basic postoperative parameters in ICU** | |
| Need for postoperative catecholamines | 0 (0) |
| Need for postoperative ventilation | 0 (0) |
| Enteral nutrition | 0 (0) |
| Length of stay in ICU | 0 (0) |
| Length of stay in hospital | 0 (0) |
| **Daily scores and clinical characteristics at admission** | |
| SAPS II (score) | 0 (0) |
| SOFA (score) | 0 (0) |
| Urine production (ml/24h) | 1 (1.2) |
| **Arterial laboratory values at admission** | |
| pO_2_ (mmHg) | 13 (15.5) |
| pCO_2_ (mmHg) | 1 (1.2) |
| pH | 1 (1.2) |
| Bicarbonate (mmol/l) | 2 (2.4) |
| Base excess (mmol/l) | 1 (1.2) |
| Lactate (mmol/l) | 1 (1.2) |
| **Venous laboratory values at admission** | |
| Hemoglobin (g/dl) | 2 (2.4) |
| Leukocyte count (x10^9^/l) | 2 (2.4) |
| Thrombocyte count (x10^9^/l) | 2 (2.4) |
| Creatinine (mg/dl) | 2 (2.4) |
| Bilirubin (mg/dl) | 5 (6.0) |
| Aspartate transaminase (AST) (U/l) | 46 (54.8) |
| Alanine transaminase (ALT) (U/l) | 10 (11.9) |
| Gamma-glutamyl transferase (GGT) (U/l) | 55 (65.5) |
| Lactate dehydrogenase (LDH) (U/l) | 64 (76.2) |
| C-reactive protein (CRP) (mg/l) | 11 (13.1) |
| International normalized ratio (INR) | 8 (9.5) |

**Supplementary table 1**. Number and percentage of missing values per parameter

| Variables | Total  N (%) / median (IQR)  (N=26) | Survival > 28 days  N (%) / median (IQR) (N=13) | Exitus within 28 days  N (%) / median (IQR) (N=13) | P value  Univariate comparison |
| --- | --- | --- | --- | --- |
| **Parameters of mechanical ventilation** | | | | |
| Duration of mechanical ventilation (days) | 0.455  (0.236-1.1125) | 0.409  (0.236-1.29) | 0.49  (0.42-1) | 0.798 |
| PEEP at ICU admission (cmH_2_O) | 5.9  (5.2-7.1) | 5.2  (5.0-6.2) | 7  (5.6-8.0) | 0.025 |
| FiO_2_ at ICU admission (%) | 37.5 (30-50) | 35 (30-40) | 50 (35-50) | 0.049 |
| Respiratory rate at ICU admission (1/min) | 17 (14-20) | 15 (14-20) | 19 (14-20) | 0.807 |

**Supplementary table 2.** Parameters of patients that were admitted with mechanical ventilation divided by survival status.

| Variables | Total  N (%)  (N=84) |
| --- | --- |
| **Pre-operative surgical diagnosis** | |
| Ileus (ref: no) | 30 (35.7%) |
| Carcinoma (ref: no) | 26 (30.9%) |
| Bleeding (ref: no) | 14 (16.7%) |
| Cholecystitis (ref: no) | 11 (13.1%) |
| Perforation (ref: no) | 11 (13.1%) |
| Ischemia (ref: no) | 10 (11.9%) |
| Trauma (ref: no) | 6 (7.1%) |
| Anal prolapse (ref: no) | 3 (3.6%) |
| Hemothorax (ref: no) | 3 (3.6%) |
| Sigma diverticulitis (ref: no) | 3 (3.6%) |
| Appendicitis (ref: no) | 2 (2.4%) |
| Metastasis (ref: no) | 1 (1.2%) |
| Struma multinodosa (ref: no) | 1 (1.2%) |

**Supplementary table 3.** Most common pre-operative surgical diagnosis.

| Variables | Total  N (%)  (N=84) |
| --- | --- |
| **Intraoperative procedures** | |
| At least one intestinal anastomosis (ref: no) | 24 (28.6%) |
| Colon resection (ref: no) | 24 (28.6%) |
| Cholecystectomy (ref: no) | 17 (20.2%) |
| Laparoscopy (ref: no) | 17 (20.2%) |
| Small intestine resection (ref: no) | 13 (15.5%) |
| Stoma operation (ref: no) | 11 (13.1%) |
| Hernia surgery (ref: no) | 9 (10.7%) |
| Overstitching of perforation (ref: no) | 7 (8.3%) |
| Adhesiolysis with adhesive ileus (ref: no) | 6 (7.1%) |
| Appendectomy (ref: no) | 6 (7.1%) |
| Laparotomy without further intervention (ref: no) | 4 (4.8%) |
| Stomach resection (ref: no) | 4 (4.8%) |
| VATS/Thoracotomy (ref: no) | 3 (3.6%) |
| Discontinuity resection (ref: no) | 2 (2.4%) |
| Liver resection (ref: no) | 2 (2.4%) |
| Catheter-implantation (ref: no) | 1 (1.2%) |

**Supplementary table 4.** Most common intraoperative procedures performed.

| 24h after admission | Physiological range according to [16] and [17] | Total  N (%) / median (IQR)  (N=82) | Survival > 28 days  N (%) / median (IQR) (N=57) | Exitus within 28 days  N (%) / median (IQR) (N=25) | P value  Univariate comparison |
| --- | --- | --- | --- | --- | --- |
| **Daily scores and clinical characteristics 24h after admission** | | | | | |
| SOFA (score) |  | 2 (1-4) | 1 (0-3) | 4 (2-10) | <0.0001 |
| Need for catecholamines (ref:no) |  | 26 (30.9%) | 11 (19.3%) | 15 (55.6%) | 0.001 |
| Need for ventilation (ref:no) |  | 12 (14.6%) | 5 (41.7%) | 7 (58.3%) | 0.023 |
| Urine production (ml/24h) |  | 710 (280-1040) | 710 (250-980) | 600 (340-1250) | 0.697 |
| **Arterial laboratory values 24h after admission** | | | | | |
| pO_2_ (mmHg) | 80-100 | 86.1 (73-108) | 86.1 (73-102) | 87.4 (64.3-108) | 0.694 |
| pCO_2_ (mmHg) | 35-45 | 38 (32.8-41.9) | 39.2 (34.35-42.05) | 36.5 (29.7-39.4) | 0.089 |
| pH | 7.38-7.44 | 7.41 (7.38-7.45) | 7.41 (7.39-7.45) | 7.36 (7.31-7.44) | 0.008 |
| Bicarbonate (mmol/l) | 21-28 | 23.65 (21.6-25.9) | 24.3 (23.1-25.9) | 21.1 (17.3-24.1) | 0.0005 |
| Base excess (mmol/l) | -4-+2 | -0.8 (-3.9-1.8) | -0.3 (-1.9-2.6) | -4 (-9.1--0.5) | 0.0007 |
| Lactate (mmol/l) | 0.6-1.7 | 1 (0.7-1.5) | 0.9 (0.6-1.1) | 1.3 (1-1.8) | 0.0009 |
| **Venous laboratory values 24h after admission** | | | | | |
| Hemoglobin (g/dl) | 12.0-17.5 | 10.3 (9.1-11.2) | 10.3 (9.1-11.2) | 10.4 (9.1-11.1) | 0.553 |
| Leukocyte count (x10^9^/l) | 4.5-11.0 | 11 (8.0-14.1) | 10.9 (8.0-14.1) | 11.1 (7.3-13.7) | 0.709 |
| Thrombocyte count (x10^9^/l) | 150-350 | 224 (171-264.5) | 228 (181-266) | 191 (148-252) | 0.185 |
| Creatinine (mg/dl) | <1.5 | 0.92 (0.7-1.48) | 0.88 (0.70-1.20) | 1.35 (0.96-1.91) | 0.001 |
| Bilirubin (mg/dl) | 0.3-1.0 | 0.6 (0.4-1) | 0.6 (0.4-0.9) | 0.7 (0.4-1) | 0.355 |
| Aspartate transaminase (AST) (U/l) | 0-35 | 35 (19-54) | 33 (18-47) | 45 (22-110) | 0.323 |
| Alanine transaminase (ALT) (U/l) | 0-35 | 15.5 (11-30) | 15 (10-26) | 23 (12-31) | 0.135 |
| Gamma-glutamyl transferase (GGT) (U/l) | 1-94 | 45 (35-195.5) | 35 (24-40) | 196 (100-319) | 0.013 |
| Lactate dehydrogenase (LDH) (U/l) | 100-190 | 210 (169-313) | 210 (169-212) | 296 (161-3075) | 0.462 |
| C-reactive protein (CRP) (mg/l) | 0.08-3.10 | 91 (51-149) | 88 (51-125) | 98 (72-153) | 0.426 |
| International normalized ratio (INR) | 0.8-1.1 | 1.1 (1.0-1.2) | 1.1 (1.0-1.2) | 1.2 (1.1-1.3) | 0.041 |

**Supplementary table 5.** Laboratory values and scores 24 hours after admission stratified by survival status.

| 48h after admission | Physiological range according to [16] and [17] | Total  N (%) / median (IQR)  (N=42) | Survival > 28 days  N (%) / median (IQR) (N=26) | Exitus within 28 days  N (%) / median (IQR) (N=16) | P value  Univariate comparison |
| --- | --- | --- | --- | --- | --- |
| **Daily scores and clinical characteristics 48h after admission** | | | | | |
| SOFA (score) |  | 2 (1-4) | 2 (1-3) | 4 (2-7) | 0.069 |
| Urine production (ml/24h) |  | 950 (570-1990) | 940 (585-1775) | 990 (450-1959) | 0.884 |
| **Arterial laboratory values 48h after admission** | | | | | |
| pO_2_ (mmHg) | 80-100 | 84.9 (69.95-95.2) | 83.2 (68.55-89.6) | 85.6 (76.1-109.5) | 0.403 |
| pCO_2_ (mmHg) | 35-45 | 41.1 (33.6-44.4) | 41.7 (35.6-45.1) | 36.5 (30.9-42.5) | 0.132 |
| pH | 7.38-7.44 | 7.41 (7.36-7.44) | 7.42 (7.39-7.44) | 7.38 (7.31-7.45) | 0.304 |
| Bicarbonate (mmol/l) | 21-28 | 24.2 (22.6-25.8) | 24.55 (23.6-26.2) | 22.1 (19.7-24.5) | 0.009 |
| Base excess (mmol/l) | -4-+2 | -0.4 (-2.2-1.7) | 0.7 (-1.2-2.4) | -3 (-6.2-0.2) | 0.011 |
| Lactate (mmol/l) | 0.6-1.7 | 0.9 (0.7-1.4) | 0.8 (0.7-1.2) | 1.1 (0.9-1.5) | 0.048 |
| **Venous laboratory values 48h after admission** | | | | | |
| Hemoglobin (g/dl) | 12.0-17.5 | 9.45 (8.4-10.4) | 9.5 (8.4-10.4) | 9.1 (8-10.9) | 0.969 |
| Leukocyte count (x10^9^/l) | 4.5-11.0 | 11.25 (9.5-14.2) | 10.7 (9.1-14) | 11.8 (10.1-15) | 0.300 |
| Thrombocyte count (x10^9^/l) | 150-350 | 208 (162-263) | 206 (164-257) | 214 (150-334) | 0.818 |
| Creatinine (mg/dl) | <1.5 | 1.17 (0.8-1.7) | 0.99 (0.80-1.40) | 1.69 (0.85-2.20) | 0.025 |
| Bilirubin (mg/dl) | 0.3-1.0 | 0.4 (0.3-0.6) | 0.4 (0.2-0.5) | 0.4 (0.3-0.6) | 0.129 |
| Aspartate transaminase (AST) (U/l) | 0-35 | 30 (17-46) | 25.5 (14-37) | 47 (30-68) | 0.091 |
| Alanine transaminase (ALT) (U/l) | 0-35 | 16 (12-22) | 16 (11-22) | 13 (12-22) | 0.924 |
| C-reactive protein (CRP) (mg/l) | 0.08-3.10 | 164 (111-197) | 168 (121-192) | 151 (101-202) | 0.588 |
| International normalized ratio (INR) | 0.8-1.1 | 1.1 (1.08-1.21) | 1.1 (1.1-1.2) | 1.2 (1.1-1.2) | 0.096 |

**Supplementary Table 6.** Laboratory values and scores 48 hours after admission stratified by survival status. LDH and GGT values were omitted since they consisted of less than 10 datapoints.

| Non-survival after 365 days | Coefficient | 95% confidence interval | | P-Value |
| --- | --- | --- | --- | --- |
| **Sociodemographics, health, and preexisting medical conditions** | | | | |
| Living situation before admission | 1.05 | 0.45 | 2.44 | 0.915 |
| Limitation of therapy | 23.69 | 2.88 | 194.82 | 0.003 |
| Preexisting cardiac disease | 4.86 | 1.04 | 22.68 | 0.045 |
| **Clinical characteristics at admission** | | | | |
| Need for postoperative catecholamines | 1.33 | 0.17 | 10.60 | 0.785 |
| Need for postoperative ventilation | 2.66 | 0.17 | 40.45 | 0.481 |
| Enteral nutrition | 0.48 | 0.04 | 5.53 | 0.559 |
| SAPS II (score) | 1.06 | 0.99 | 1.13 | 0.090 |
| SOFA (score) | 0.62 | 0.38 | 1.01 | 0.056 |
| Urine production (ml/24h) | 1.00 | 1.00 | 1.00 | 0.095 |
| **Arterial laboratory values at admission** | | | | |
| pH | 0.57 | 0.00 | 1351559.95 | 0.941 |
| Lactate (mmol/l) | 1.78 | 0.74 | 4.29 | 0.195 |
| Base excess (mmol/l) | 1.13 | 0.90 | 1.42 | 0.299 |
| **Venous laboratory values at admission** | | | | |
| Creatinine (mg/dl) | 4.98 | 1.12 | 22.13 | 0.035 |
| International normalized ratio (INR) | 196.92 | 0.74 | 52722.63 | 0.064 |
| Model Estimator | Number of observations = 73  Likelihood Ratio= 35.22  Prob > chi2 = 0.0014 | | | |

**Supplementary table 7.** Logistic regression analysis of selected variables divided by survival status after 365 days corrected for significant baseline.
